# Health care use and costs of adverse drug events emerging from outpatient treatment in Germany: A modelling approach

**DOI:** 10.1186/1472-6963-11-9

**Published:** 2011-01-13

**Authors:** Renee G Stark, Jürgen John, Reiner Leidl

**Affiliations:** 1 Institute for Health Economics and Health Care Management, Helmholtz Zentrum München - German Research Center for Environmental Health, Ingolstädter Landstraße 1, Neuherberg, 85764, Germany; 2 Department of Health Economics and Health Care Management in the Munich School of Management at the University of Munich, Ludwigstr. 28, Munich, 80539, Germany

## Abstract

**Background:**

This study's aim was to develop a first quantification of the frequency and costs of adverse drug events (ADEs) originating in ambulatory medical practice in Germany.

**Methods:**

The frequencies and costs of ADEs were quantified for a base case, building on an existing cost-of-illness model for ADEs. The model originates from the U.S. health care system, its structure of treatment probabilities linked to ADEs was transferred to Germany. Sensitivity analyses based on values determined from a literature review were used to test the postulated results.

**Results:**

For Germany, the base case postulated that about 2 million adults ingesting medications have will have an ADE in 2007. Health care costs related to ADEs in this base case totalled 816 million Euros, mean costs per case were 381 Euros. About 58% of costs resulted from hospitalisations, 11% from emergency department visits and 21% from long-term care. Base case estimates of frequency and costs of ADEs were lower than all estimates of the sensitivity analyses.

**Discussion:**

The postulated frequency and costs of ADEs illustrate the possible size of the health problems and economic burden related to ADEs in Germany. The validity of the U.S. treatment structure used remains to be determined for Germany. The sensitivity analysis used assumptions from different studies and thus further quantified the information gap in Germany regarding ADEs.

**Conclusions:**

This study found costs of ADEs in the ambulatory setting in Germany to be significant. Due to data scarcity, results are only a rough indication.

## Background

Medications are used to cure or slow disease processes, to reduce symptoms and to improve quality of life [1]. However, all medications may have disadvantageous effects, which may be reported as drug related problems (DRPs) or adverse drug events (ADEs). Studies of DRPs report actual or potential problems which interfere with the desired health outcome, a spectrum ranging from adverse consequences (such as side effects) to lack of effectiveness [2]. In contrast, studies of ADEs report injuries due to the use of a drug [3]. ADEs may be due to: 1) medication errors; 2) adverse drug reactions (ADRs), i.e. unintended reactions occurring at usual doses [4]; 3) interactions with other drugs, underlying diseases or the patient (idiosyncratic reactions and allergies) or 4) errors in prescribing, dispensing, adhering to and monitoring medications [1,5].

Literature reviews regarding the number of hospital admissions due to ADRs have reported various results. One review in 2002 reported that ADRs account for 4.9% of hospital admissions [6] whereas another review in 1997 reported that 5.8% of all admissions to medical departments [4] were drug-related. Only 2 studies have reported drug-related hospitalisations to internal medicine wards in Germany. Dormann [7] reported that 3.8% of medical admissions were drug-related, while Schneeweiss reported that 2.4% of all medical admissions over 30 months were drug-related [8].

The proportion of preventable ADEs is significant [9], ranging from 3.7% (range 1.4-15.4) if all hospital admissions are considered [10] to 30.7% if only admissions to medical department are considered [11]. Preventable drug-related admissions were associated with prescribing problems (30.6%), adherence problems (33.3%) and monitoring problems (22.2%) [10].

In US emergency departments, 1/3 of ADEs treated in persons over 65 were caused by warfarin, insulin and digoxin, all having a narrow therapeutic index and a high risk of overdose or toxicity [12]. Similarly in Germany, antithrombotics, NSAIDs, insulin, salicylates, digoxin and calcium antagonists have been reported to account for 70% of the medications involved in drug-related hospitalisations [8]. Medications responsible for preventable drug-related admissions include antiplatelets (50%), aspirin (16%), diuretics (15.9%), non-steroidals (11%) and anticoagulants (8.3%) [10].

It is estimated that in the USA, ADEs occur at a rate of 2-7/100 admissions in hospital [9] and at a rate of 3% in adult primary care outpatients [13], thus imposing a considerable burden on healthcare systems. A review of the international literature regarding costs of ADEs from the hospital perspective reported that average hospital costs ranged from 904€ to 5,783€ per ADE with both the lowest and highest values reported in the USA [14]. Annual hospitalisation costs for ADEs in Germany, were estimated, based on a literature review, to total 1,050 million DM (540 million Euros) in 1997 [11]. Since 30% of these adverse events were possibly preventable, at least 180 million Euros were unnecessary costs [11]. However, these calculations did not consider outpatient treatment costs of ADEs.

Costs associated with drug-related mortality and morbidity in ambulatory care have been estimated using a probability-pathway model for the USA [15,16]. The model is based on probabilities of resource use estimated by clinical experts for the US healthcare system. It identifies and structures the possible resource use related to adverse drug events occurring in the ambulatory setting. Estimates of costs associated with drug-related morbidity and mortality exceeded $177.4 billion US dollars for the year 2000, with hospital admissions accounting for nearly 70% of total costs. This equates to 13% of the total US expenditures for health in 2000 (1,328 billion US dollars according to OECD data [17]).

### Aim

Previous studies for Germany have reported the percent of hospitalisations due to ADRs and the associated costs for the hospital admission [7,8]. However, the burden of morbidity, mortality and costs of ADEs originating and occurring in ambulatory medical practice remains unknown. It was our objective to estimate morbidity and costs associated with adverse drug events occurring in the ambulatory setting in Germany. To estimate the frequency and costs of ADEs occurring in the context of outpatient drug treatment, the drug-related mortality and morbidity cost-of-illness model developed for the US was adapted to incorporate available German data. Sensitivity analyses were performed using rates of ADEs and healthcare utilisation found in the literature.

## Methods

### Model to determine the frequency and costs of ADEs

**Figure 1:**
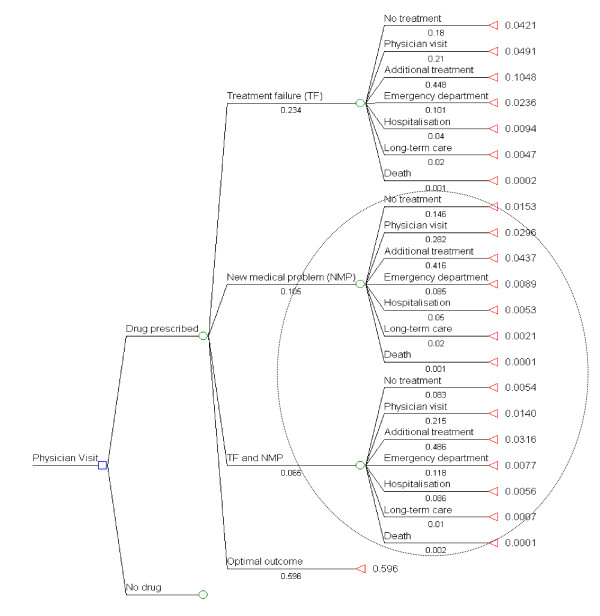
**Probabilities of morbidity and mortality associated with medication intake used in the modelling approach**. Enclosed in the oval is the part of the model used for the scenario. Probability of drug prescription in the original model was 0.55, and no drug was 0.45. Overall outcome probabilities obtained by multiplying probability of type of drug-related problem with probability of outcome of drug-related problem (probabilities originate from the model published by Ernst et al., 2001 [17]).

Parts of the drug-related morbidity and mortality cost-of-illness-model [15,16] were used to simulate the morbidity and costs of ADEs in Germany. This model describes outcomes and costs associated with drug-related problems (DRPs) from the perspective of third party payers but it excludes illicit drug use and DRPs originating in institutional settings. In the model, drug related problems are divided into three mutually exclusive sequences of events: 1) treatment failure 2) new medical problem (NMP) 3) treatment failure and new medical problem (TF/NMP). The structure of the model is shown in Figure 1. The probability of these sequences of events, or that 'no DRP occurs' and the conditional probabilities of healthcare utilisation if a DRP developed were estimated by a panel of clinical experts for the USA [15,16]. In the model, the healthcare utilisation endpoint is mostly mutually exclusive, i.e. if the patient is hospitalised, costs for previous consultations to the physician for the DRP are not considered and for long-term care, no prior hospitalisation or physician visit is included. This results in a systematic downward bias in the costing of these healthcare resources. In the case of death, the cost of prior hospitalisation is included as a cost of death. However, one can imagine that some patients with a severe ADE will die before reaching the hospital. Since the aim of this study is to estimate the frequency and costs of ADEs and the study used as a source of ADEs [8] did not document treatment failures, only the sequences of events for NMP and TF/NMP are included in the present calculations, the part of the model enclosed by a circle in Figure 1.

In the paper by Ernst et al. [16], the number of ADEs is calculated based on a cohort of patients making a total number of 734,493,000 physician visits. Since 90% of the German population is insured with social health insurance, healthcare facilities are used more frequently than in the USA. There were 7.4 physician visits per capita in Germany in 2006 compared to 3.8 in the USA according to OECD [17]. Similarly, prescription practices between Germany and the USA may differ considerably. However, it is conceivable that the likelihood of requiring healthcare services, such as hospitalisation, will be similar between the Germany and the USA if an ADE occurs. Thus, only the event probabilities after the node "drug prescribed" will be used for the present calculations. Our final probabilities are the result of multiplying the first node (NMP or TF/NMP) with the probability of resource utilisation. For example, in the paper by Ernst et al. [16], the final probability of "no therapy" for a "new medical problem" was 0.633 × 0.105 × 0.146 = 0.010. In our paper, we did not include the probability of drug therapy in the calculation (0.633), so that the probability of "No therapy" for a "new medical problem" is 0.0153. Our adapted probabilities are shown in Figure 1. As hospitalisations are included in the model as hospitalisations and death, the total number of hospitalisations is divided between these categories, 97.9% as hospitalisations and 2.1% as death. The probabilities of the types of resource utilisation (physician visit, additional treatment, emergency department visit, hospitalisation, long-term care and death) of the 2 model arms, as shown in Figure 1 were added together to obtain overall probabilities for the various types of resource utilisation for ADEs. Overall probabilities of resource use for ADEs were that 12% of medication users will require a physician visit (4% only consultation and 8% additional treatment), 2% an emergency department visit, 1% a hospitalisation, 0.3% long-term care and 0.02% will die.

### Sources of Costs of Resource Utilisation

To calculate the hospital costs of ADEs in the year 2007, the adverse drug reactions described in the Schneeweiss paper [8] were assigned possible ICD 10 codes (3 positions). Using data from the Federal Office of Statistics for patients over 20 years of age, the sum of hospital days reported for the possible ICD codes for each described adverse drug reaction was divided by the total number of patients with the ICD code to calculate an average length of stay. The calculated average length of stay was multiplied with the average cost per hospital day for general hospitals, 437€ [18]. The ICD codes of the adverse drug reactions, the number of patients per ICD code, number of hospital days per ICD code and the average cost per admission per adverse drug reaction is shown in Additional File 1. The final cost of hospital stay was calculated as the average cost of the described adverse drug reaction weighted according to its prevalence as reported by Schneeweiss [8]. The assignment of adverse drug reactions, their proportion in each drug class and the calculated weights and total costs are shown in Additional File 2. The final calculated cost of hospital stay (3,452.11€) was the weighted average cost of the described adverse drug reactions. Hospital admission costs are also included in the costs of death. The costs of other resource utilisation were determined as follows. The cost of an emergency department visit was assumed to equal the cost of one day in hospital (437€) [18]. The average cost of a prescribed medication for the German statutory health insurance system, which covers about 90% of the resident population, was 42.62 Euro in 2007 [19]. The cost of a physician visit was calculated based on the average contact value of a general practitioner and specialists of internal medicine with and without family practice responsibilities [20] in 1999 (16.20€ in 1999). The contact value was extrapolated to 2007 using price increases in physician reimbursement per case [21], which averaged 4.4% annually between 1999 and 2007. The value for 2007 was calculated to be 22.62 Euro per visit. Average costs for long-term care [22] in 2007 were calculated by dividing total expenditures for long term care in the year 2007 [23] (18.34 billion Euros) by the total number of recipients receiving benefits [24] (2.03 million). This average includes persons receiving benefits for the whole year or less, but it was assumed that the majority of persons would receive benefits for the whole year. This value was divided by 12 to obtain the monthly rate. The time points at which persons started to require long-term care for an ADE were assumed to be equally distributed across the year and among the persons requiring long-term care. Since expenditures were not reported according to age-groups, the overall average was used, which amounted to 4,890 Euro. Multiple visits to any healthcare facility were not accounted for since these data were not available for Germany. This cost calculation aimed to determine the excess costs of ADEs and thus the costs of initial treatment were not included, since they occur regardless of a ADE.

### Source of the Number of Hospitalisations due to ADEs

Since the only available data for Germany is the percent of hospitalisations related to adverse drug events [7,8], the model was adapted to be based on this data. The relationship between the probabilities is used to calculate resource utilisation from the number of ADEs requiring hospitalisation (ie. 4 times as many patients require only a physician visit compared to those requiring hospitalisation, the calculated factors are shown in Table 1). The number of hospitalisations due to ADEs were calculated based on the Schneeweiss study [8], whereas the Dormann study [7] was used in the sensitivity analysis. Schneeweiss reported that 2.4% of all hospital admissions to medical wards over 30 months in 2 German cities (total of 3 hospitals) were drug-related leading to an estimate of the expected number of drug-related admissions of adults of 139,405 based on the published number of admissions to medical wards [25] (5,808,544 admissions in 2007) for the ages 20 and older. All other health resource use was calculated in relationship to the number of drug-related hospitalisations.

**Table 1:** Annual resource use of the base case, resource unit costs and total costs of drug-related problems associated with the outcomes of drug therapy according to probabilities of the model and population numbers

	**U.S. Data from Ernst et al. 2001 model **[16]		German Data (Base Case)		
**Outcomes**	Overall probability of therapeutic outcome*	**Factor in relation to hospitalisation**	Expected population developing an ADE and classification of resource utilization** (as % of population ingesting medications)	**Unit cost (in 2007 €)**	**Total costs (million €)**

No treatment	0.0207	1.912	260,873 (0.5)		
Physician visit	0.0436	4.021	548,620 (1.1)	23^1^	12.4
Additional treatment	0.0753	6.944	947,451 (1.9)	42^2^+23^1 ^(65^3^)	61.8
Emergency department	0.0166	1.531	208,887 (0.4)	437^4^	91.3
Hospital admissions	0.0108	1.000	136,447 (0.3)	3452^5^	471.0
Long-term care	0.0028	0.254	34,615 (0.07)	4890^6^	169.3
Death	0.0002	0.022	2,958 (0.006)	3452^7^	10.2

Total costs					816.0

*sum of probabilities associated with therapeutic outcomes in Figure 1 **calculated using the factor in relation to hospitalisation and the number of hospitalised persons; ^1^average cost of published physician visits [20] inflated to year 2007; ^2^average cost of a prescribed medication in 2007 [19]^3^Total cost of additional treatment comprises physician visit and prescription of a new medication ^4^average cost per hospital day [18]; ^5^average cost per hospital admission for an ADE in 2007 (calculated by authors); ^6^average cost of 6 months of long-term facility care in 2007 (total service expenditures [23] divided by total beneficiaries [24]; ^7^costs of death assume hospitalisation prior to death

### Sensitivity Analyses

In order to test estimates based on the model, MEDLINE was searched for studies reporting the frequency of ADEs in adults in the ambulatory setting published after 1990. Studies including chemotherapeutic agents or only certain diseases or specific ADEs were excluded. The studies found in this search are listed and summarized in Additional File 3. Rates of healthcare resource use reported by the studies for outpatient services (e.g. general practitioner, clinic), use of the emergency department, and hospitalisations are shown in Table 2. The table shows the reported rates of healthcare use and adjusted rates of healthcare use according to the reported population taking medications (see also Additional File 3), since prescription rates may vary between countries.

**Table 2:** Range of healthcare utilisation according to outpatient literature

Healthcare facilities used for ADE/ADR	Healthcare utilisation per ADE/ADR in %	Healthcare utilisation according to the population ingesting medications in %
Physician visit	48.2 [36] - 88.7 [37]	3.5 [28] - 22.2 [29]
Emergency care	8.6 [29] - 15.7 [28]	0.9 [28] - 3.0 [29]
Hospitalisation	4.0 [38] - 12.4 [37]	0.50 [28] - 4.19 [29]

ADE: adverse drug event; ADR: adverse drug reaction; Study references are in square brackets

The following sensitivity analyses were performed. To test the effect of a higher rate of drug-related hospitalisations, the complete model was calculated based on the rate reported by Dormann [7]. Other one-way sensitivity analyses examined upper and lower rates of specific resource use (physician visits, emergency department visits and hospitalisations) for ADEs, as reported in the studies found (Table 2). In these sensitivity analyses, the absolute numbers determined for base case healthcare utilisation (Schneeweiss study) were applied and only the numbers for the specific resource examined (e.g. physician visits) were changed. The rates of healthcare resource used from the literature review, adjusted according to the population treated with medications in the study (see Table 2) are multiplied with the population treated with medications in Germany. The estimate of the German population taking medications is based on the percent of health insurance company members, according to age, who had a medication prescribed in 2007 [26]. Percentages were adjusted for differences in age and sex-distribution between the health insurance population and the general population [27]. Overall, about 75% of the German population over 20 years of age (53.6 million of 66.2 million persons) take at least one prescribed medication over the year. All calculations were performed using Microsoft Excel.

## Results

### Base Case

The base case calculations using German data are shown in Table 1. The base case estimates that 2.14 million adults would develop ADEs during the course of ambulatory medical treatment in 2007. Only 12% of these patients require no extra healthcare services and 70% require only outpatient services such as a physician visit or additional outpatient treatment. In relation to the total German population taking medications, about 4.4% of the population ingesting medications in 2007 would be expected to have an ADE and about 3.8% would be expected to require health services. 3.0% would require ambulatory medical care (physician visit and additional treatment), 0.3% hospitalisation and 0.006% would die.

Expected resource use and cost estimates associated with ADEs are also reported in Table 1 (costs of resource utilisation and sources of costs are also shown in the table). According to the model, ADEs may have accounted for 816 million Euros of health care expenditures in Germany over 1 year, with 80% of expenditures for hospitalisation (also preceding death) and long-term care being generated by 8% of the predicted patients with ADEs. Ambulatory care, without the emergency department, accounts for 9% of all costs but is required by 70% of patients. Since 12% of patients with ADEs generate no costs, the average cost of an ADE requiring health services is 381 Euro if all persons with an ADE are considered and 434 Euro if only those seeking healthcare are considered.

### Sensitivity Analysis

The sensitivity analyses in which the German hospitalisation rate [7] or only the rates of physician visits, emergency department visits or hospitalisation individually are varied, are shown in Table 3. All rates are based on the rates reported in the literature (Table 2) adjusted according to the study population taking medications. The sensitivity analysis (Table 3) shows that the estimates based on the German hospitalisation rate reported by Schneeweiss [8] are much lower than the other estimates of the frequency and costs of ADEs. The calculation using Dormann's hospitalisation rate has a strong effect on both the number of ADEs and the costs postulated since the complete model is recalculated. Since the base case probabilities and resource costs are used, the cost per case is equal to that of Schneeweiss (381€). Dormann's percentage of hospitalisations due to ADRs is still lower than other values found in the literature. Variation of the number of physician visits has a large effect on the number of ADEs but a small effect on the total costs and leads to lower costs per case (upper limit: 117€ per case; lower limit: 358€ per case) than estimated by Schneeweiss. Variation of the number of hospitalisations has the greatest effect on the overall costs of ADEs. The upper limit of the number of hospitalisations estimates total costs which are 9 times higher than in the base case and costs per case which are almost 5 times higher than costs per case of the base case.

**Table 3:** Sensitivity analysis of variables contributing to the costs of drug-related morbidity and mortality model

	Sensitivity analyses	Limit	Description of rate used*	Resulting Parameter Size	Resulting ADEs (thousands)	Resulting costs of ADEs (million €)
1.	Comparison of effect of German hospitalisation rates on model	Lower (base case)	Schneeweiss [8]: 2.4% of medical admissions	Hospitalisations**: 139,405	2,140	816
		Upper	Dormann [7] : 3.8% of medical admissions	Hospitalisations:	3,388	1,292
				Total: 220,724		
				(Death: 4,684)		
2.	Variation in rate of physician visits according to literature***	Lower	3.5% [28] (total = 1,731,649)	Physician visits: 635,008	2,375	828
				Additional treatment: 1,096,640		
		Upper	22% [29] (total = 11,075,753)	Physician visits: 4,061,560	11,719	1,291
				Additional treatment: 7,014,193		
3.	Variation in emergency department visits according to literature	Lower	0.9% [28]	432,912	2,364	914
		Upper	3.0% [29]	1,499,696	3,431	1,380
4.	Change in only hospital admissions according to literature	Lower	0.5% [28]	Hospitalisations: 246,603	2,252	1,205
				Deaths: 5346		
		Upper	4.2% [29]	Hospitalisations: 2,051,109	4,096	7,569
				Deaths: 44,466		

*Rates refer to percentage of patients taking medications unless stated otherwise; **Equal to base case calculations; ***1/3 calculated as only physician visit and 2/3 rds with additional treatment)

## Discussion

This study uses a probability pathway model to estimate the frequency and costs of ADEs occurring in ambulatory medical practice in Germany over 1 year (2007). The model includes drug-related morbidity and mortality. It calculates ADEs and bases all resource utilisation on the number of hospital admissions attributed to ADEs reported by the German Schneeweiss study [8]. Results indicate that about 2 million ADEs have occurred in persons in the population over 20 years of age taking medications, and that subsequent health care for 816 million Euros has been utilized.

The predicted probabilities of ADEs and resource utilisation due to ADEs in the population treated with medications were lower for the German base case than in the original model, but close to the lower end of rates reported in the literature, indicating a limited usefulness of the model in this respect. The calculation of total costs of resource utilisation attributes the highest proportion of costs to hospitalisation, the factor in the model for which there are the most data for Germany. Thus, in this respect, the model is useful in estimating the lower limits of resource utilisation costs which can be expected to be due to ADEs. According to the base case, hospital admission due to an ADE is required by 0.3% of the population taking medications (see Table 1). In comparison, the other German study predicts 0.4% and the original NMP and TF/NMP arms of the model together predict 1.1%. The studies of ADEs in ambulatory medical practice observed that between 0.5% [28] and 4.2% [29] of the population taking medications required hospitalisation due to an ADE (see Table 2). These studies indicate that the base case likely underestimates the total number of ADEs, whereas the model rates are likely too high for Germany. The original model rates regarding mortality due to ADEs, 2.1% of patients hospitalised for ADEs, are also probably too high for Germany, since Schneeweiss reported a rate of 1.7% and Dormann a rate of about 1%.

There are no German studies regarding other types of resource utilisation for ADEs, such as physician or emergency visits. However, the base case predicts rates in the treated population of both physician visits (3%) and emergency visits (0.4%) which are lower than those predicted using Dormann's study (5% and 0.7% respectively), the original model (12% and 2% respectively) and close to the lower end of rates reported in the literature (3.5% and 0.9% respectively: see Table 2). Basing the model on German hospitalisation rates to predict the rates of ADEs in the outpatient population leads to estimates of ADEs lower than reported in the literature. According to the base case, 4.3% of the treated population will have an ADE, whereas if Dormann's values are used, 6.9% of the treated population will have an ADE. In the literature, probabilities for ADEs range between 5.5% [28] and 34.7% [29] of the treated population (see Additional File 3).

As already implied above, this study has numerous limitations. First, both studies used for the German data are limited. The Schneeweiss study [8] reports admission rates due to ADRs which are considerably lower than other studies but only non-elective admissions were evaluated and patients with skin reactions were not included. Also, the 3 hospitals in the study may not have been representative for usual medical practice in Germany. A follow-up study with intensified ADR surveillance in these hospitals, reported a higher ADR incidence rate of 3.25% [30] while using the original exclusion criteria. The second German study was based on admissions to a university hospital, which may have a higher probability of ADE admissions. This study is also limited by a smaller number of cases, a shorter observation period, a baseline population which included readmissions and transfers from other wards and hospitals and the inclusion of ADRs due to chemotherapeutic agents. Of further concern is that the origin of patients in both German studies is not described, thus whether they were living independently or in a nursing home is unknown. Also, both German studies only report ADRs, a subgroup of ADEs. The gap between the estimates for the base case and sensitivity analyses (Table 3) also point out the gap in the available information for Germany and the possible number of unrecognised ADEs. The sensitivity analyses are also limited, since all studies of ADEs in the ambulatory population were performed in the USA and thus may not reflect the incidence or resource use in Germany. Due to its simple structure, the model does not reflect all costs possibly incurred by ADEs, such as multiple physician visits, multiple hospitalisations, increased costs of health services around the time of death [31-34], or other costly resources such as long-term dialysis for specific ADEs [35]. However, the use of a Markov model would seem inappropriate at the present, since the literature provided neither exact treatment sequences for ADEs, nor rates of recovery from ADEs.

Our estimations for the ambulatory sector show the importance of hospital and long-term care costs, which account for 80% of ADE costs but are generated by only 8% of patients predicted to have an ADE. In contrast, costs of physician visits and additional treatment explain 9% of total ADE costs but are generated by 70% of patients predicted to have an ADE. The overall costs reported by Ernst et al. [16] are considerably higher than our estimates, but their calculations also include costs for treatment failure, the most frequent drug-related problem in their model. Generally, their cost structure is similar, with hospitalisations and long-term care accounting for 87% of all costs, while physician visits and additional prescriptions account for 9.8% of costs.

Overall, the costs predicted by the sensitivity analyses were much higher than the base case, indicating an underestimation of true costs. However, all studies used for the sensitivity analyses originated in the USA and reflect the US population and health care system. This attempt at transferring probabilities from a model developed for the USA to Germany, illustrates the extent of required information which is still missing such as prescribing practices, the rate of medication changes per patient, estimates of compliance and the classes of medications prescribed. The absence of universal healthcare coverage in the US may have numerous consequences on health care utilisation, such as rate of purchase of prescribed medications, how closely patients are monitored regarding medications and whether patients seek medical advice if symptoms occur. The corresponding behaviour and habits of patients and physicians in Germany must be considered to decide how well these studies and rates apply in Germany.

Considering the limitations of the model and the parameters used, the postulated costs of 816 million Euros (0.32% of German healthcare expenditures in 2007 (253 billion Euros) [17]), due to ADEs must be viewed with caution. They represent first attempts for Germany to postulate costs associated with ADEs emerging from outpatient treatment and vividly illustrate the lack of available data and difficulties in assessing any results. Although outpatient research of ADEs may be difficult in Germany, due to data confidentiality and the separation of in- and outpatient care, estimates based on the model show that adverse drug events may be placing a significant economic burden on the healthcare system in Germany, especially in terms of hospital and long-term care costs. As the population ages, the disease burden will increase, more medications will be consumed and the risk of severe adverse events and hospitalisation will increase [3]. Investigations of outpatient ADEs in Germany could determine the types of ADEs occurring and useful prevention processes. At present, any calculations assessing the benefits or costs related to processes aiming at reducing adverse drug events, such as an electronic health card, would require much more data than available in the published literature in this area.

## Conclusions

ADEs pose a significant problem in outpatient treatment which may be preventable by improving communication between pharmacists, physicians and patients. Studies assessing the frequency of ADEs in the ambulatory setting exist for the USA but were not found for Germany. Costs related to ADEs in the ambulatory setting were postulated using a cost-of-illness model. A major proportion of these costs were attributed to hospitalisation and to long-term care. Due to the paucity of information regarding the ambulatory setting, it was difficult to assess whether cases, resource use and costs were valid for Germany. As all estimates in the sensitivity analysis were higher, base case results can be considered a very conservative estimate. To more precisely estimate the population-related costs in Germany, reliable data would be required on the prevalence of ADEs in general as well as for specific medications and the share of ADEs which are preventable.

## Competing interests

The authors declare that they have no competing interests.

## Authors' contributions

RS participated in the design of the study, performed the literature review, performed statistical analyses and drafted the manuscript. JJ participated in its design and helped to draft the manuscript. RL participated in its design and coordination and helped to draft the manuscript. All authors read and approved the final manuscript.

## Pre-publication history

The pre-publication history for this paper can be accessed here:

http://www.biomedcentral.com/1472-6963/11/9/prepub

## Supplementary Material

Additional file 1 **Mean hospital costs calculated for each adverse drug reaction described by Schneeweiss**. All possible ICD codes which are associated with an adverse drug reaction as described in the Schneeweiss paper were determined using the German DRG grouper. Using data from the Federal Office of Statistics for patients over 20 years of age, the sum of hospital days reported for the possible ICD codes for each described adverse drug reaction was divided by the total number of patients with the ICD code to calculate an average length of stay. The calculated average length of stay was multiplied with the average cost per hospital day for general hospitals, 437€ [18]. Click here for file

Additional file 2 **Mean overall hospital costs calculated using mean costs of adverse drug reactions and weighting these according to their frequency of occurrence in each drug class as described in the paper by Schneeweiss**. Mean overall weighted hospital costs were calculated using the average hospital cost per adverse drug reaction (ADR), shown in additional file 1 and weighting these costs according to the frequency of these adverse drug reactions in the drug classes listed in the Schneeweiss paper and then according to the proportion of the drug classes as a total of all ADRs (also in the Schneeweiss paper). Click here for file

Additional file 3 **Literature review of studies examining the frequency of adverse drug events in ambulatory patients**. MEDLINE was searched for studies reporting the frequency of adverse drug events (ADEs) in adults in the ambulatory setting published after 1990. Studies including chemotherapeutic agents or only certain diseases or specific ADEs were excluded. The studies found in this search are listed and summarized regarding their methodology and results. Click here for file
