# Supplementary material for: Health care use and costs of adverse drug events emerging from outpatient treatment in Germany: A modelling approach
Source: BMC Health Serv Res. 2011 Jan 13;11:9. doi: 10.1186/1472-6963-11-9 (PMC3032652; doi:10.1186/1472-6963-11-9)
Supplement: Additional file 1 — Mean hospital costs calculated for each adverse drug reaction described by Schneeweiss. All possible ICD codes which are associated with an adverse drug reaction as described in the Schneeweiss paper were determined using the German DRG grouper. Using data from the Federal Office of Statistics for patients over 20 years of age, the sum of hospital days reported for the possible ICD codes for each described adverse drug reaction was divided by the total number of patients with the ICD code to calculate an average length of stay. The calculated average length of stay was multiplied with the average cost per hospital day for general hospitals, 437€ [18]. [file 1472-6963-11-9-S1.DOC]

Additional File 1: Mean hospital costs calculated for each adverse drug reaction described by Schneeweiss

| Adverse drug reaction | ICD 10 | Description of ICD 10 Code | Number of persons admitted to hospital^1^ | Number of hospital admission days^2^ | Mean length of admission^3^ (Days) | Cost of average admission^4^ |
| --- | --- | --- | --- | --- | --- | --- |
|  |  |  |  |  |  |  |
| Gastroduodenal bleeding | K25 | Gastric ulcer | 50292 | 485745 |  |  |
|  | K26 | Duodenal ulcer | 28814 | 290170 |  |  |
|  | K28 | Gastrojejunal ulcer | 1223 | 12938 |  |  |
|  | K29 | Gastritis and duodenitis | 96093 | 539398 |  |  |
|  | K31 | Other diseases of stomach and duodenum | 12623 | 111963 |  |  |
|  | I85 | Oesophageal varices | 3077 | 24299 |  |  |
|  | K92 | Other diseases of digestive system | 39872 | 261292 |  |  |
|  | K27 | Peptic ulcer, site unspecified | 634 | 6065 | 7.44 | 3253.38 |
|  |  |  |  |  |  |  |
| Gastroduodenal lesions | K25 | Gastric ulcer | 50292 | 485745 |  |  |
|  | K26 | Duodenal ulcer | 28814 | 290170 |  |  |
|  | K29 | Gastritis and duodenitis | 96093 | 539398 |  |  |
|  | K27 | Peptic ulcer, site unspecified | 634 | 6065 |  |  |
|  | K28 | Gastrojejunal ulcer | 1223 | 12938 |  |  |
|  | K31 | Other diseases of stomach and duodenum | 12623 | 111963 |  |  |
|  | K92 | Other diseases of digestive system | 39872 | 261292 |  |  |
|  | K52 | Other noninfective gastroenteritis and colitis | 75664 | 440672 | 7.04 | 3075.81 |
|  |  |  |  |  |  |  |
| Gastrointestinal hemorrhage | K25 | Gastric ulcer | 50292 | 485745 |  |  |
|  | K26 | Duodenal ulcer | 28814 | 290170 |  |  |
|  | K28 | Gastrojejunal ulcer | 1223 | 12938 |  |  |
|  | K29 | Gastritis and duodenitis | 96093 | 539398 |  |  |
|  | K31 | Other diseases of stomach and duodenum | 12623 | 111963 |  |  |
|  | I85 | Oesophageal varices | 3077 | 24299 |  |  |
|  | K27 | Peptic ulcer, site unspecified | 634 | 6065 |  |  |
|  | K55 | Vascular disorders of intestine | 18340 | 210066 |  |  |
|  | K92 | Other diseases of digestive system | 39872 | 261292 | 7.74 | 3381.41 |
|  |  |  |  |  |  |  |
| Diarrhea | K52 | Other noninfective gastroenteritis and colitis | 94252 | 485246 |  |  |
|  | K58 | Irritable bowel syndrome | 7554 | 38956 |  |  |
|  | K59 | Other functional intestinal disorders | 33684 | 168069 | 5.55 | 2426.61 |
|  |  |  |  |  |  |  |
| Vomiting | R11 | Nausea and vomiting | 11923 | 58938 | 4.94 | 2160.19 |
|  |  |  |  |  |  |  |
| Hepatitis | K71 | Toxic liver disease | 5099 | 50041 |  |  |
|  | K72 | Hepatic failure, not elsewhere classified | 4031 | 48705 |  |  |
|  | K75 | Other inflammatory liver diseases | 5498 | 64606 |  |  |
|  | T50 | Poisoning by diuretics and other and unspecified drugs, medicaments and biological substances | 8962 | 25282 | 8.00 | 3494.41 |
|  |  |  |  |  |  |  |
| Urticaria / anaphylaxis | T88 | Other complications of surgical and medical care, not elsewhere classified | 13652 | 60393 |  |  |
|  | T78 | Adverse effects, not elsewhere classified | 22286 | 58327 |  |  |
|  | L50 | Urticaria | 9788 | 41999 |  |  |
|  | T39 | Poisoning by nonopioid analgesics, antipyretics and antirheumatics | 2881 | 6786 | 3.68 | 1608.30 |
|  |  |  |  |  |  |  |
| Bradycardia | R00 | Abnormalities of heart beat | 15718 | 75728 | 4.82 | 2105.43 |
|  |  |  |  |  |  |  |
| Bradycardia / Atrioventricular block | I44 | Atrioventricular and left bundle-branch block | 24946 | 217491 |  |  |
|  | R00 | Abnormalities of heart beat | 15718 | 75728 | 7.21 | 3151.11 |
|  |  |  |  |  |  |  |
| Syncope | R55 | Syncope and collapse | 134004 | 784905 |  |  |
|  | I95 | Hypotension | 31115 | 175876 | 5.82 | 2542.78 |
|  |  |  |  |  |  |  |
| Atrioventricular block | I44 | Atrioventricular and left bundle-branch block | 24946 | 217491 | 8.72 | 3809.97 |
|  |  |  |  |  |  |  |
| Stoke-Adams syndrome | I45 | Other conduction disorders | 6285 | 43367 | 6.90 | 3015.33 |
|  |  |  |  |  |  |  |
| Torsades de pointes | I47 | Paroxysmal tachycardia | 52127 | 308085 | 5.91 | 2582.79 |
|  |  |  |  |  |  |  |
| Other arrhythmias | I45 | Other conduction disorders | 6285 | 43367 |  |  |
|  | I47 | Paroxysmal tachycardia | 52127 | 308085 |  |  |
|  | I48 | Atrial fibrillation and flutter | 207922 | 1331382 |  |  |
|  | I49 | Other cardiac arrhythmias | 46502 | 400415 |  |  |
|  | R00 | Abnormalities of heart beat | 15718 | 75728 | 6.57 | 2871.59 |
|  |  |  |  |  |  |  |
|  |  |  |  |  |  |  |
| Hypotension | R55 | Syncope and collapse | 134004 | 784905 |  |  |
|  | R57 | Shock, not elsewhere classified | 6697 | 42318 |  |  |
|  | I95 | Hypotension | 31115 | 175876 | 5.84 | 2551.30 |
|  |  |  |  |  |  |  |
| Hypoglycemia in diabetics and non-diabetics | E15 | Nondiabetic hypoglycaemic coma | 713 | 5048 |  |  |
|  | E16 | Other disorders of pancreatic internal secretion | 2943 | 16826 |  |  |
|  | E11 | Non-insulin-dependent diabetes mellitus | 174155 | 2285874 |  |  |
|  | E13 | Other specified diabetes mellitus | 2952 | 31061 |  |  |
|  | E14 | Unspecified diabetes mellitus | 2873 | 32412 |  |  |
|  | E10 | Insulin-dependent diabetes mellitus | 22636 | 220374 | 12.56 | 5490.45 |
|  |  |  |  |  |  |  |
|  |  |  |  |  |  |  |
| Hypoglycemia due to insulin | E11 | Non-insulin-dependent diabetes mellitus | 174155 | 2285874 |  |  |
|  | E13 | Other specified diabetes mellitus | 2952 | 31061 |  |  |
|  | E14 | Unspecified diabetes mellitus | 2873 | 32412 |  |  |
|  | E10 | Insulin-dependent diabetes mellitus | 22636 | 220374 |  |  |
|  | T38 | Poisoning by hormones and their synthetic substitutes and antagonists, not elsewhere classified | 455 | 1894 | 12.68 | 5542.35 |
|  |  |  |  |  |  |  |
| Hypoglycemia (NIDDM) | E11 | Non-insulin-dependent diabetes mellitus | 174155 | 2285874 |  |  |
|  | E13 | Other specified diabetes mellitus | 2952 | 31061 |  |  |
|  | E14 | Unspecified diabetes mellitus | 2873 | 32412 | 13.05 | 5704.33 |
|  |  |  |  |  |  |  |
| Hypoglycemia -only diabetics / Diabetes | E11 | Non-insulin-dependent diabetes mellitus | 174155 | 2285874 |  |  |
|  | E13 | Other specified diabetes mellitus | 2952 | 31061 |  |  |
|  | E14 | Unspecified diabetes mellitus | 2873 | 32412 |  |  |
|  | E10 | Insulin-dependent diabetes mellitus | 22636 | 220374 | 12.68 | 5542.35 |
|  |  |  |  |  |  |  |
| Hyperkalemia | E87 | Other disorders of fluid, electrolyte and acid-base balance | 14970 | 131245 | 8.77 | 3831.27 |
|  |  |  |  |  |  |  |
| Electrolyte disturbances | E87 | Other disorders of fluid, electrolyte and acid-base balance | 14970 | 131245 | 8.77 | 3831.27 |
|  |  |  |  |  |  |  |
|  |  |  |  |  |  |  |
| Dehydration or dizziness | E86 | Volume depletion | 59386 | 446016 |  |  |
|  | H81 | Disorders of vestibular function | 58122 | 327789 |  |  |
|  | H82* | Vertiginous syndromes in diseases classified elsewhere | 0 | 0 |  |  |
|  | R42 | Dizziness and giddiness | 30228 | 159877 | 6.32 | 2761.81 |
|  |  |  |  |  |  |  |
| Agranulocytosis / pancytopenia (aplastic anemia) | D70 | Agranulocytosis | 7416 | 52701 |  |  |
|  | D61 | Other aplastic anaemias | 6105 | 47019 |  |  |
|  | D60 | Acquired pure red cell aplasia [erythroblastopenia] | 278 | 2391 | 7.40 | 3233.75 |
|  |  |  |  |  |  |  |
| Intracerebral / intracranial bleed | I60 | Subarachnoid haemorrhage | 11334 | 193413 |  |  |
|  | I61 | Intracerebral haemorrhage | 33623 | 527029 |  |  |
|  | I62 | Other nontraumatic intracranial haemorrhage | 7297 | 88847 | 15.49 | 6768.08 |
|  |  |  |  |  |  |  |
| Other bleeding | D68 | Other coagulation defects | 11671 | 100050 |  |  |
|  | R31 | Unspecified haematuria | 10003 | 52273 |  |  |
|  | R58 | Haemorrhage, not elsewhere classified | 838 | 5566 |  |  |
|  | R04 | Haemorrhage from respiratory passages | 18493 | 77991 |  |  |
|  | N93 | Other abnormal uterine and vaginal bleeding | 2814 | 9955 | 5.61 | 2451.67 |
|  |  |  |  |  |  |  |
| Anemia | D50 | Iron deficiency anaemia | 31949 | 242126 |  |  |
|  | D62 | Acute posthaemorrhagic anaemia | 6308 | 53520 |  |  |
|  | D63* | Anaemia in chronic diseases classified elsewhere | 0 | 0 |  |  |
|  | D64 | Other anaemias | 14452 | 103266 | 7.57 | 3307.30 |
|  |  |  |  |  |  |  |
| Thrombocytopenia | D69 | Purpura and other haemorrhagic conditions | 6862 | 64838 | 9.45 | 4129.15 |
|  |  |  |  |  |  |  |
| Deep vein thrombosis / thrombophlebitis | I80 | Phlebitis and thrombophlebitis | 3077 | 24299 | 7.90 | 3450.98 |
|  |  |  |  |  |  |  |
|  |  |  |  |  |  |  |
| Osteoporosis | M80 | Osteoporosis with pathological fracture | 23152 | 298854 |  |  |
|  | M81 | Osteoporosis without pathological fracture | 4147 | 41722 | 12.48 | 5451.91 |
|  |  |  |  |  |  |  |

Note: ADR: adverse drug reaction

^1^ Total number of persons 20 years or older admitted to hospital for the ICD code according to the German Federal bureau of statistics

^2^ Total number of hospital admission days for persons 20 years or older for the ICD code according to the German Federal bureau of statistics

^3^ Mean admission days determined by dividing the sum of admission days for an ADR by the number of persons with an ADR

^4^ Cost of average admission calculated by multiplying average length of stay with the average cost per hospital day for general hospitals, 437€ [18] to determine the average cost per adverse drug reaction
