# Supplementary material for: Health care use and costs of adverse drug events emerging from outpatient treatment in Germany: A modelling approach
Source: BMC Health Serv Res. 2011 Jan 13;11:9. doi: 10.1186/1472-6963-11-9 (PMC3032652; doi:10.1186/1472-6963-11-9)
Supplement: Additional file 2 — Mean overall hospital costs calculated using mean costs of adverse drug reactions and weighting these according to their frequency of occurrence in each drug class as described in the paper by Schneeweiss. Mean overall weighted hospital costs were calculated using the average hospital cost per adverse drug reaction (ADR), shown in additional file 1 and weighting these costs according to the frequency of these adverse drug reactions in the drug classes listed in the Schneeweiss paper and then according to the proportion of the drug classes as a total of all ADRs (also in the Schneeweiss paper). [file 1472-6963-11-9-S2.DOC]

Additional File 1: Mean overall hospital costs calculated using mean costs of adverse drug reactions and weighting these according to their frequency of occurrence in each drug class as described in the paper by Schneeweiss.

| Drugs Class | Number of ADR Cases per Drug Class | Proportion of all ADRs accounted for by the Drug Class^1^ | Number of ADR Cases per Drug type | Drug Types | Number of Cases per ADR | Proportion of each ADR in a Drug Class^2^ | Adverse drug reaction | Weight^3^ | Mean Cost of the ADR^4^ | Weighted cost^5^ | Total cost^6^ |
| --- | --- | --- | --- | --- | --- | --- | --- | --- | --- | --- | --- |
| Analgesics and antirheumatics | 267 | 0.219 |  |  |  |  |  |  |  |  |  |
|  |  |  | 168 | NSAIDs | 95 | 0.397 | Gastroduodenal bleeding | 0.087 | 3253.38 | 283.48 |  |
|  |  |  |  |  | 41 | 0.172 | Gastroduodenal lesions | 0.038 | 3075.81 | 115.67 |  |
|  |  |  |  |  | 5 | 0.021 | Diarrhea | 0.005 | 2426.61 | 11.13 |  |
|  |  |  |  |  | 5 | 0.021 | Urticaria/anaphylaxis | 0.005 | 1608. 30 | 7.38 |  |
|  |  |  |  |  | 3 | 0.013 | Agranulocytosis/ pancytopenia | 0.003 | 3233.75 | 8.90 |  |
|  |  |  |  |  |  |  |  |  |  |  |  |
|  |  |  | 99 | Salicylates | 57 | 0.238 | Gastroduodenal bleeding | 0.052 | 3253.38 | 170.09 |  |
|  |  |  |  |  | 26 | 0.109 | Gastroduodenal lesions | 0.024 | 3075.81 | 73.35 |  |
|  |  |  |  |  | 4 | 0.017 | Urticaria/anaphylaxis | 0.004 | 1608.30 | 5.90 |  |
|  |  |  |  |  | 3 | 0.013 | Hypoglycemia | 0.003 | 5490.45 | 15.11 | 691.00 |
|  |  |  |  |  |  |  |  |  |  |  |  |
|  |  |  |  |  | 239 | 1.000 |  |  |  |  |  |
|  |  |  |  |  |  |  |  |  |  |  |  |
| Cardiovascular drugs | 450 | 0.369 |  |  |  |  |  |  |  |  |  |
|  |  |  | 96 | Digoxin/ digitoxin | 31 | 0.084 | Bradycardia. | 0.031 | 2105.43 | 65.35 |  |
|  |  |  |  |  | 20 | 0.054 | Syncope. | 0.020 | 2542.78 | 50.92 |  |
|  |  |  |  |  | 6 | 0.016 | AV block. | 0.006 | 3809.97 | 22.89 |  |
|  |  |  |  |  | 6 | 0.016 | Stoke-Adams syndrome. | 0.006 | 3015.33 | 18.11 |  |
|  |  |  |  |  | 3 | 0.008 | Torsades de pointes | 0.003 | 2582.79 | 7.76 |  |
|  |  |  |  |  | 9 | 0.024 | Other arrhythmia | 0.009 | 2871.59 | 25.88 |  |
|  |  |  |  |  | 5 | 0.014 | Vomiting | 0.005 | 2160.19 | 10.81 |  |
|  |  |  |  |  |  |  |  |  |  |  |  |
|  |  |  | 91 | Calcium antagonists | 12 | 0.033 | Syncope | 0.012 | 2542.78 | 30.55 |  |
|  |  |  |  |  | 9 | 0.024 | Bradycardia. | 0.009 | 2105.43 | 18.97 |  |
|  |  |  |  |  | 4 | 0.011 | Stoke-Adams syndrome. | 0.004 | 3015.33 | 12.08 |  |
|  |  |  |  |  | 8 | 0.022 | Other arrhythmia | 0.008 | 2871.59 | 23.00 |  |
|  |  |  |  |  | 29 | 0.079 | Gastroduodenal bleeding | 0.029 | 3253.38 | 94.47 |  |
|  |  |  |  |  | 6 | 0.016 | Gastroduodenal lesions | 0.006 | 3075.81 | 18.48 |  |
|  |  |  |  |  | 7 | 0.019 | Hypotension | 0.007 | 2551.30 | 17.88 |  |
|  |  |  |  |  |  |  |  |  |  |  |  |
|  |  |  | 73 | ACE inhibitors | 15 | 0.041 | Hypoglycemia | 0.015 | 5542.35 | 83.24 | : |
|  |  |  |  |  | 12 | 0.033 | Syncope | 0.012 | 2542.78 | 30.55 |  |
|  |  |  |  |  | 3 | 0.008 | Bradycardia | 0.003 | 2105.43 | 6.32 |  |
|  |  |  |  |  | 6 | 0.016 | Other arrhythmia | 0.006 | 2871.59 | 17.25 |  |
|  |  |  |  |  | 10 | 0.027 | Hypotension | 0.010 | 2551.30 | 25.54 |  |
|  |  |  |  |  | 3 | 0.008 | Hyperkalemia | 0.003 | 3831.27 | 11.51 |  |
|  |  |  |  |  |  |  |  |  |  |  |  |
|  |  |  | 73 | Beta blockers | 15 | 0.041 | Syncope | 0.015 | 2542.78 | 38.19 |  |
|  |  |  |  |  | 17 | 0.046 | Bradycardia | 0.017 | 2105.43 | 35.84 |  |
|  |  |  |  |  | 8 | 0.022 | Stoke-Adams syndrome | 0.008 | 3015.33 | 24.15 |  |
|  |  |  |  |  | 4 | 0.011 | AV block | 0.004 | 3809.97 | 15.26 |  |
|  |  |  |  |  | 8 | 0.022 | Other arrhythmia | 0.008 | 2871.59 | 23.00 |  |
|  |  |  |  |  | 13 | 0.035 | Hypoglycemia | 0.013 | 5542.35 | 72.14 | : |
|  |  |  |  |  | 5 | 0.014 | Hypotension | 0.005 | 2551.30 | 12.77 |  |
|  |  |  |  |  |  |  |  |  |  |  |  |
|  |  |  | 50 | Diuretics | 13 | 0.035 | Syncope | 0.013 | 2542.78 | 33.10 |  |
|  |  |  |  |  | 12 | 0.033 | Electrolyte disturbances | 0.012 | 3831.27 | 46.03 |  |
|  |  |  |  |  | 5 | 0.014 | Hypotension | 0.005 | 2551.30 | 12.77 |  |
|  |  |  |  |  | 5 | 0.014 | Bradycardia/ AV block | 0.005 | 3151.11 | 15.78 |  |
|  |  |  |  |  | 3 | 0.008 | Dehydration or dizziness | 0.003 | 2761.81 | 8.30 |  |
|  |  |  |  |  |  |  |  |  |  |  |  |
|  |  |  | 43 | Nitrates | 8 | 0.022 | Hypotension | 0.008 | 2551.30 | 20.44 |  |
|  |  |  |  |  | 10 | 0.027 | Syncope | 0.010 | 2542.78 | 25.46 |  |
|  |  |  |  |  | 7 | 0.019 | Bradycardia | 0.007 | 2105.43 | 14.76 |  |
|  |  |  |  |  | 4 | 0.011 | Stoke-Adams syndrome | 0.004 | 3015.33 | 12.08 |  |
|  |  |  |  |  | 4 | 0.011 | Other arrhythmia | 0.004 | 2871.59 | 11.50 |  |
|  |  |  |  |  | 7 | 0.019 | Gastroduodenal lesions | 0.007 | 3075.81 | 21.56 |  |
|  |  |  |  |  |  |  |  |  |  |  |  |
|  |  |  | 14 | Other antihypertensives | 4 | 0.011 | Syncope | 0.004 | 2542.78 | 10.18 |  |
|  |  |  |  |  | 3 | 0.008 | Hypotension | 0.003 | 2551.30 | 7.66 |  |
|  |  |  |  |  | 4 | 0.011 | Arrhythmia | 0.004 | 2871.59 | 11.50 |  |
|  |  |  |  |  |  |  |  |  |  |  |  |
|  |  |  | 10 | Angiotensin-2 blockers | 3 | 0.008 | Bradycardia/ AV block | 0.003 | 3151.11 | 9.47 |  |
|  |  |  |  |  | 3 | 0.008 | Syncope/ hypotension | 0.003 | 2551.30 | 7.66 | 1081.15 |
|  |  |  |  |  |  |  |  |  |  |  |  |
|  |  |  |  |  | 369 | 1.000 |  |  |  |  |  |
|  |  |  |  |  |  |  |  |  |  |  |  |
| Antidiabetics | 151 | 0.124 |  |  | |  |  |  |  |  |  |
|  |  |  | 98 | Insulin | 68 | 0.459 | Hypoglycemia | 0.057 | 5534.00 | 315.22 |  |
|  |  |  |  |  | 29 | 0.196 | Hypoglycemic coma | 0.024 | 5534.00 | 134.43 |  |
|  |  |  |  |  |  |  |  |  |  |  |  |
|  |  |  | 53 | Oral antidiabetics | 30 | 0.203 | Hypoglycemia | 0.025 | 5704.33 | 143.35 |  |
|  |  |  |  |  | 21 | 0.142 | Hypoglycemic coma | 0.018 | 5704.33 | 100.34 | 693.35 |
|  |  |  |  |  |  |  |  |  |  |  |  |
|  |  |  |  |  | 148 | 1.000 |  |  |  |  |  |
|  |  |  |  |  |  |  |  |  |  |  |  |
| Antithrombotics (cases) | 293 | 0.241 |  |  |  |  |  |  |  |  |  |
|  |  |  | 293 | Antithrombotics | 156 | 0.582 | GI hemorrhage | 0.140 | 3381.41 | 473.49 |  |
|  |  |  |  |  | 53 | 0.198 | Gastroduodenal lesions | 0.048 | 3075.81 | 146.33 |  |
|  |  |  |  |  | 8 | 0.030 | Intracerebral/ intracranial bleeds | 0.007 | 6768.08 | 48.60 |  |
|  |  |  |  |  | 37 | 0.138 | Other bleeds | 0.033 | 2451.67 | 81.42 |  |
|  |  |  |  |  | 5 | 0.019 | Hepatitis | 0.004 | 3494.41 | 15.68 |  |
|  |  |  |  |  | 3 | 0.011 | Anemia | 0.003 | 3307.30 | 8.91 |  |
|  |  |  |  |  | 3 | 0.011 | Thrombocytopenia | 0.003 | 4129.15 | 11.12 |  |
|  |  |  |  |  | 3 | 0.011 | Hypoglycemia | 0.003 | 5542.35 | 14.92 | 800.47 |
|  |  |  |  |  | 268 | 1.000 |  |  |  |  |  |
|  |  |  |  |  |  |  |  |  |  |  |  |
| Hormones | 57 | 0.0468 |  |  |  |  |  |  |  |  |  |
|  |  |  | 57 | Systemic corticosteroids | 12 | 0.267 | GI hemorrhage | 0.012 | 3381.41 | 42.20 |  |
|  |  |  |  |  | 10 | 0.222 | Gastroduodenal lesions | 0.010 | 3075.81 | 31.99 |  |
|  |  |  |  |  | 11 | 0.244 | Osteoporosis | 0.011 | 5451.91 | 62.37 |  |
|  |  |  |  |  | 5 | 0.111 | Deep vein thrombosis | 0.005 | 3450.98 | 17.94 |  |
|  |  |  |  |  | 4 | 0.089 | Thrombophlebitis | 0.004 | 3450.98 | 14.36 |  |
|  |  |  |  |  | 3 | 0.067 | Diabetes | 0.003 | 5542.35 | 17.29 | 186.14 |
|  |  |  |  |  |  |  |  |  |  |  |  |
|  |  |  |  |  | 45 | 1.000 |  |  |  |  |  |
|  |  |  |  |  |  |  |  |  |  |  |  |
| Total | 1218 |  |  |  |  |  |  |  |  |  | 3452.11 |
|  |  |  |  |  |  |  |  |  |  |  |  |

Note ADR: adverse drug reaction

^1^ Total number of ADRs due to a drug class divided by the total number of ADRs

^2^ Number of cases of an ADR divided by the total number of ADRs in a drug class

^3^ Weight is the proportion of all ADRs accounted for by the Drug Class multiplied with the proportion of each ADR in a Drug Class

^4^ Mean cost of ADR is determined according to Additional File 1

^5^ Weighted cost is the mean cost of the ADR multiplied with the weight of the ADR

^6^ Total cost shows the sum of the weighted costs per drug class and in the row “Total” for all drug classes
