# Supplementary material for: Health care use and costs of adverse drug events emerging from outpatient treatment in Germany: A modelling approach
Source: BMC Health Serv Res. 2011 Jan 13;11:9. doi: 10.1186/1472-6963-11-9 (PMC3032652; doi:10.1186/1472-6963-11-9)
Supplement: Additional file 3 — Literature review of studies examining the frequency of adverse drug events in ambulatory patients. MEDLINE was searched for studies reporting the frequency of adverse drug events (ADEs) in adults in the ambulatory setting published after 1990. Studies including chemotherapeutic agents or only certain diseases or specific ADEs were excluded. The studies found in this search are listed and summarized regarding their methodology and results. [file 1472-6963-11-9-S3.DOC]

Additional File 1: Literature review of studies examining the frequency of ADEs in ambulatory patients:

| Study | Rask (2005) [39] | Gurwitz (2003) [3] | Ghandi (2003) [40] | Honigman (2001) [29] | Ghandi (2000) [37] | Hanlon (1997) [30] | Chrischilles (1992) [41] | Schneider (1992) [38] |
| --- | --- | --- | --- | --- | --- | --- | --- | --- |
| Methodology |  |  |  |  |  |  |  |  |
| Age group | >=65 | >=65 | >18 | Mean: 47.9 | 20-75  (mean: 45.8) | >=65  high risk | >=65 | 58-97 |
| Setting | Medicare plan | Ambulatory care | Hospital and community based | Primary care | Primary care | VA medical clinic | Rural counties in east Iowa | Ambulatory care (geriatric/ medical clinic) |
| Duration of data collection | 1 year | 1 year | 3 months | 1 year | 1 year | 1 year | 1 year | 1 year |
| year completed | 2000 | 2000 | 2000 | 1996 | 1997 | Not reported | 1984 | 1988 |
| ADE /ADR | ADE | ADE | ADE | ADE | ADE | ADE | ADR | ADR |
| Population size (number taking medications) | 406 (380) | 27617 (24256) | 661 | 15665 | 2858 (2848) | 167 | 3170 (3029 (calculated from graph)) | 463 |
| Number of ADEs /ADRs | 134 | 1523 | 181 |  | 432 | 80 | 521 | 107 |
| Number of persons with ADE /ADR (as % of population taking medications) | 99 (26.1%) | 1523 (6.3%) | 162 (24.5%) | 864 (5.5%) | 432 (15.2%) | 58 (34.7%) | 318 (10.5%) | Overall: 97 (21%)  Definite: 47 (10.2%) |
| Number of preventable ADEs (as % of ADEs) | 72 contraindicated meds (54%) | 421 (28%) | 20 (11%) | (38%) | Charts: 8/64 (13%);  Patient report: (13% of 394) | (95% predictable) | NR | 30 / 47 ADRs:  ((64%) |
| Specifications regarding physician visits in paper | 78.9% of events reported to physician | NR | *23 (urgent care or other facility) | 68.8% of ADEs require an additional visit | 190 (patient report, n=394) sought medical attention | 63% of patients required phyisican contacts | 239 patients consulted physician | 86 noted by physician as having ADR |
| Number of physician visits calculated from paper (% of ADEs/ADRs) | 106 (78.9) |  | *23 (14.2%) | 594 (68.8%) | 190 (48.2%) | 37 (63%) |  | 86 (88.7%) |
| Number of visits calculated from paper (% of treated patients) |  |  | *23 (3.5%) | 594 (3.5%) | NR | 37 (22.2%) | 239 (7.9%) | 86 (18.6%) |
| Specification regarding emergency department | NR | NR | *3 patients emergency room visit | 15.7% of ADEs require multiple ambulatory or emergency department visits | NR | 10% of patients required emergency room visits | NR | NR |
| Number of emergency room visits calculated from paper (as % of ADEs/ADRs or if number of persons, as % of persons with ADE/ADR) |  |  | *3 (1.9%) | 136 (15.7%) |  | 5 (8.6%) |  |  |
| Number of calculated emergency room visits (as % of population taking medications) |  |  | *3 (0.5%) | 136 (0.9%) |  | 5 (3.0%) |  |  |
| Specification regarding hospitalisation | 4 persons reported hospitalisation | NR | NR | 79 required hospitalisation  (3.4 percent of admissions) | 3/64 (chart review) | 11% require hospitalisation | 17 patients were hospitalised due to ADR | 12 patients hospitalised due to ADR |
| Number of hospitalisations calculated from paper (as % of ADEs/ADRs or if number of persons, as % of persons with ADE/ADR) | 4 (4.0%) |  |  | 79 (9.1%) | 3 (4.7%) | 7 (12.1%) | 17 (5.3%) | 12 (12.4%) |
| Number of calculated hospitalisations (% of population taking medications) | 4 (0.98%) | NR | NR | 79 (0.5%) | NR | 7 (4.2%) | 17 (0.6%) | 12 (2.6%) |

ADE: adverse drug event; ADR: adverse drug reaction; NR: not reported; *Although these values are reported in the study, they were not included in the range; Study references are in square brackets
